# An intranasal adjuvanted, recombinant influenza A/H5 vaccine candidate induces broad priming against diverse influenza A/H5N1 virus clades in a phase I randomized trial in healthy adults

**DOI:** 10.21203/rs.3.rs-6059149/v1

**Published:** 2025-03-05

**Authors:** Meagan E. Deming, Franklin R. Toapanta, Marcela Pasetti, Hana Golding, Surender Khurana, Tarek Hamouda, Ali Fattom, Yuanyuan Liang, Sharon M. Tennant, Megan F. McGilvray, Paula J. Bernal, Jennifer J. Oshinsky, Shrimati Datta, Jasnehta Permala Booth, Lynda Coughlan, Kathleen M. Neuzil, Chad D. Costley, Karen L. Kotloff, Marcelo B. Sztein, Justin R. Ortiz

**Affiliations:** Center for Vaccine Development, University of Maryland School of Medicine; Center for Vaccine Development, University of Maryland School of Medicine; Center for Vaccine Development, University of Maryland School of Medicine; Center for Biologics Evaluation and Research (CBER), Food and Drug Administration; Center for Biologics Evaluation and Research (CBER), Food and Drug Administration; BlueWillow Biologics, Inc; BlueWillow Biologics, Inc; Center for Vaccine Development, University of Maryland School of Medicine; Center for Vaccine Development, University of Maryland School of Medicine; Center for Vaccine Development, University of Maryland School of Medicine; Center for Vaccine Development, University of Maryland School of Medicine; Center for Vaccine Development, University of Maryland School of Medicine; Center for Vaccine Development, University of Maryland School of Medicine; Center for Vaccine Development, University of Maryland School of Medicine; Center for Vaccine Development, University of Maryland School of Medicine; Center for Vaccine Development, University of Maryland School of Medicine; BlueWillow Biologics, Inc; Center for Vaccine Development, University of Maryland School of Medicine; Center for Vaccine Development, University of Maryland School of Medicine; Center for Vaccine Development, University of Maryland School of Medicine

## Abstract

We conducted a randomized, controlled phase I trial (NCT05397119) of a novel adjuvanted recombinant influenza A/H5 (A/Indonesia/05/2005, clade 2.1) hemagglutinin vaccine, administered intranasally in two doses 28 days apart at three antigen levels. Control groups received unadjuvanted recombinant H5 or formulation buffer placebo. Six months later, participants received a heterologous unadjuvanted inactivated influenza A/H5N1 (A/Vietnam/1203/2004, clade 1) vaccine intramuscularly. All vaccines were safe and well tolerated. After the primary intranasal series, serum hemagglutination inhibition and microneutralization responses were minimal. Increases in mucosal and serum IgG/IgA, serum surface plasmon resonance antibody binding, memory B cell and CD4 T cell activity, and antibody-dependent cell-mediated cytotoxicity were observed only in recipients primed intranasally with adjuvanted H5 vaccine. Following the inactivated H5N1 boost, robust responses across all immune assays, as well as microneutralization responses against diverse H5N1 clades (including currently circulating clade 2.3.4.4b), occurred in adjuvanted vaccine recipients, demonstrating successful priming and broad responses.

## INTRODUCTION

Influenza viruses cause significant health and economic harm through seasonal epidemics. Additionally, animal influenza viruses threaten food supplies and occasionally cross species barriers, causing disease and outbreaks in humans. The risk of emerging zoonotic influenza viruses with pandemic potential is exemplified by the extensive spread of clade 2.3.4.4b H5N1 avian influenza viruses in poultry and livestock, with sporadic cases of human illness^1^.

Current intramuscular influenza vaccines induce strain-specific systemic immune responses to the major surface glycoprotein, hemagglutinin (HA). These responses effectively prevent symptomatic illness when vaccines are well-matched to circulating strains but may be less effective at preventing infection^2,3^. In contrast, mucosal vaccines, which stimulate immune responses at the site of infection, may provide superior protection against both viral shedding and onward transmission of influenza^3^. Recognizing this potential, public health organizations have advocated for improved mucosal vaccines, particularly those capable of broadening immunity against diverse influenza viruses^4,5^.

For traditional intramuscular influenza vaccines, a hemagglutination inhibition (HAI) titer ≥40 is considered a relative correlate of protection^6^ and serves as a standard for licensure of seasonal and pandemic influenza vaccines^7,8^. In contrast, mucosal influenza vaccine development faces challenges due to the lack of established immune correlates of protection^9^. Only one mucosal influenza vaccine, a live-attenuated influenza vaccine (LAIV), is licensed in the United States^9^. This vaccine often does not meet the regulatory HAI standard^9^, and its clinical development required extensive field trials to demonstrate efficacy^10^. Developing mucosal vaccines for emerging pathogens like avian influenza faces even greater challenges. For instance, a 2007 clinical trial of an H5N1 LAIV failed to elicit HAI responses^11^; however, participants who later received an intramuscular H5N1 boost exhibited significant immune recall^12–15^, a pattern observed with other avian influenza LAIVs followed by intramuscular boosts^14^. For mucosal avian influenza vaccines, defining correlates of protection would be valuable in advancing products through clinical development.

To address these challenges, we conducted a phase I randomized controlled trial of a novel adjuvanted intranasal influenza A/H5 subtype vaccine. We assessed product safety and performed extensive immunologic analyses to explore markers of mucosal vaccine immune priming.

## RESULTS

### Clinical trial design and participants.

There were five vaccine groups. Three groups received clade 2.1 influenza A/H5 (A/Indonesia/05/2005) recombinant hemagglutinin glycoprotein (rH5)^16^ at one of three dose levels (25 μg, 50 μg, and 100 μg) combined with an oil-in-water nanoemulsion (NE) adjuvant^17,18^; one group received unadjuvanted rH5 (100 μg); and one group received placebo. Vaccines were administered intranasally on Days 1 and 29. Six months later (Day 197), all participants received a heterologous intramuscular boost with an unadjuvanted 90 μg dose of a licensed, inactivated clade 1 influenza A/H5N1 (A/Vietnam/1203/2004) vaccine (H5N1 IIV)^19^.

**Figure 1:**
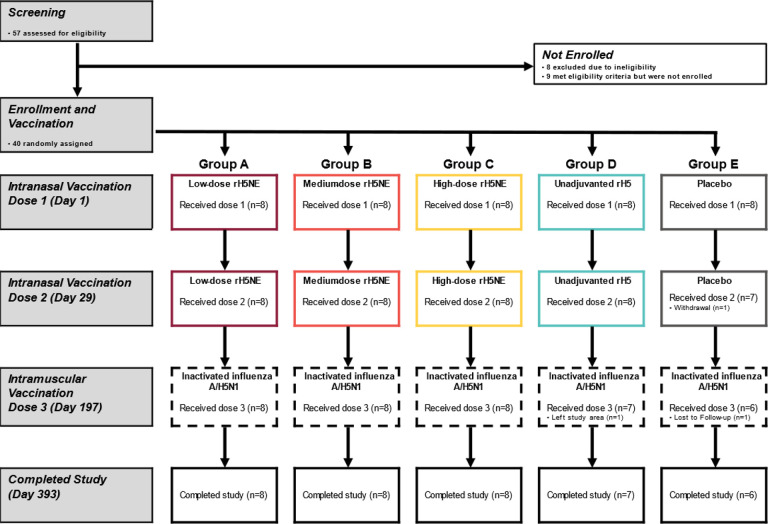
Consort diagram Forty participants were enrolled and received the first vaccination. The withdrawal of one participant (Group E) before the second vaccination was not related to any safety event. A second participant (Group D) left the study area after receiving the second vaccination and could not complete the trial. A third participant (Group E) did not receive the third vaccination and was lost to follow up. He did not respond to multiple inquiries from the study team, but his emergency contact informed the study team that the participant was well.

The trial was conducted from July 7, 2022, through October 12, 2023. Forty healthy adults aged 18–45 years were enrolled and randomized, with eight participants each assigned to Group A (low-dose rH5-NE), Group B (medium-dose rH5-NE), Group C (high-dose rH5-NE), Group D (unadjuvanted high-dose rH5), and Group E (placebo) (Fig. 1).

Overall, 45% of participants were women, 18% were Black or African American, and 15% were of Hispanic ethnicity. Their mean age was 30.2 years. Participant demographic and baseline information by vaccine group are in **Online display 1.**

### Hemagglutination inhibition (HAI) responses.

**Figure 2:**
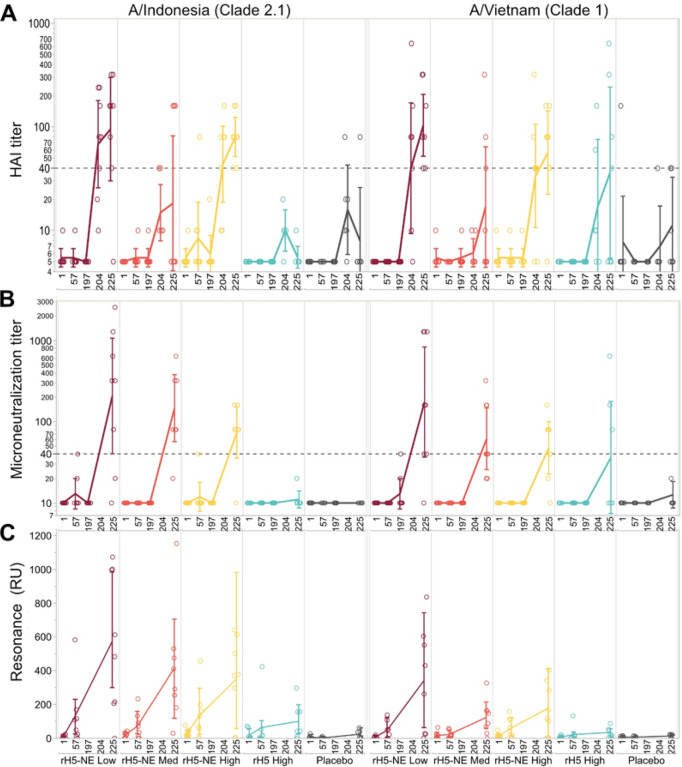
Hemagglutination inhibition, microneutralization, and surface plasmon resonance by vaccine strain and group Antibody responses to influenza A/H5N1 A/Indonesia/05/2005 (clade 2.1) and A/Vietnam/1203/2004 (clade 1) at baseline, Day 57 (28 days post-second intranasal vaccination), Day 197 (immediately before intramuscular H5N1 IIV boost), and Day 225 (28 days post intramuscular IIV boost). Individual serum HAIs (**A**) and microneutralization (**B**) titers are shown with lines connecting the geometric mean titers at each timepoint with 95% confidence intervals. Individual Surface Plasmon Resonance (SPR) responses shown with connecting mean and standard deviations (**C**). HAI assays were also conducted with specimens from Day 204 (seven days post intramuscular IIV boost). For the HAI assay, sera that were negative at the initial dilution were assigned a titer of 5. For the MN assay, sera that were negative at the initial dilution were assigned a titer of 10. Dotted lines in panels **A** and **B** show the 1:40 dilution. Low-dose (Group A), medium-dose (Group B) and high-dose (Group C) of rH5-NE are shown in maroon, orange and yellow. Controls, including the unadjuvanted rH5 (Group D) and placebo (Group E) are shown in cyan and gray.

Baseline immunity by HAI Geometric Mean Titer (GMT) to rH5 (clade 2.1) and H5N1 IIV (clade 1) was low among all groups (GMT ≤ 5.5), and there were no significant increases on Days 57 or 197 after intranasal vaccinations (Fig. 2 **panel A** and **Online display 2).** Four weeks after H5N1 IIV was administered, we observed significant GMT responses among Group A and Group C compared to baseline or Day 57 (p < 0.05). Geometric mean fold rise (GMFR) to rH5 (clade 2.1) at Day 225 among the rH5-NE groups were 17.4 (95% CI 4.6, 66.0) for Group A, 3.7 (95% CI 0.82, 16.4) for Group B, and 14.7 (95% CI 10.1, 21.3) for Group C, while the GMFR at Day 225 remained low for comparator groups, 1.1 (95% CI 0.9, 1.4) for Group D and 1.6 (95% CI 0.5, 5.2) for Group E. The percentage of participants with seroconversion at Day 225 to rH5 (clade 2.1) was 87.5% (95% CI 47.3, 99.7) for Group A, 37.5% (95% CI 8.5, 75.5) for Group B, 100.0% (95% CI 63.1, 100.0) for Group C, 0.0% (95% CI 0.0, 41.0) for Group D, and 16.7% (95% CI 0.0, 64.1) for Group E.

Day 225 HAI GMTs responses were significantly higher than baseline or Day 57 (p < 0.05) for Group A and Group C against H5N1 IIV (clade 1), with the highest titers among the rH5-NE groups (Fig. 2 **panel A** and **Online display 2).** GMFR to H5N1 IIV (clade 1) at Day 225 were 20.7 (95% CI 10.4, 41.3) for Group A, 3.1 (95% CI 0.9, 11.0) for Group B, 10.4 (95% CI 3.9, 27.5) for Group C, 7.2 (95% CI 1.1, 48.6) for Group D, and 2.2 (95% CI 0.8, 6.5) for Group E. The percentage of participants with seroconversion at Day 225 to H5N1 IIV (clade 1) were 100.0% (95% CI 63.1, 100.0) for Group A, 37.5% (95% CI 8.5, 75.5) for Group B, 87.5% (95% CI 47.3, 99.7) for Group C, 57.1% (95% CI 18.4, 90.1) for Group D, and 33.3% (95% CI 4.3, 77.7) for Group E.

In a post-hoc analysis, we conducted HAI to rH5 (clade 2.1) and H5N1 IIV (clade 1) at Day 204, seven days after the intramuscular H5N1 IIV administration (Fig. 2 **panel A** and **Online display 2**). The GMFR results at Day 204 were similar to the Day 225 values for both H5N1 clades, suggesting a recall response rather than a primary immune response to the intramuscular vaccination.

### Microneutralization (MN) responses.

Similar to HAI results, we measured no increase in MN titers on Days 57 or 197 to either A/Indonesia/05/2005 (clade 2.1) or H5N1 A/Vietnam/1203/2004 (clade 1), and we observed significant boosted immune responses at Day 225 compared to baseline and Day 57 among the rH5-NE groups to both strains (Fig. 2 **panel B and Online display 3**). MN GMFR to A/Indonesia/05/2005 (clade 2.1) at Day 225 were 20.7 (95% CI 1.0, 106.7) for Group A, 14.7 (95% CI 5.7, 38.0) for Group B, 7.3 (95% CI 3.6, 15.1) for Group C, 1.1 (95% CI 0.9, 1.4) for Group D, and 1.0 (95% CI 1.0, 1.0) for Group E. The percentage of participants with seroconversion at Day 225 to A/Indonesia/05/2005 (clade 2.1) was 75% (95% CI 34.9, 96.8) for Group A, 87.5% (95% CI 47.3, 99.7) for Group B, 87.5% (95% CI 47.3, 99.7) for Group C, 0.0% (95% CI 0.0, 41.0) for Group D, and 0.0% (95% CI 0.0, 45.9) for Group E.

MN GMFR to H5N1 A/Vietnam/1203/2004 (clade 1) at Day 225 were 17.5 (95% CI 3.7, 83.2) for Group A, 6.2 (95% CI 2.6, 14.8) for Group B, 4.8 (95% CI 2.3, 10.0) for Group C, 3.6 (95% CI 0.7, 17.8) for Group D, and 1.3 (95% CI 0.9, 1.8) for Group E (Fig. 2 **panel B and Online display 3**). The percentage of participants with seroconversion at Day 225 to H5N1 A/Vietnam/1203/2004 (clade 1) was 87.5% (95% CI 47.3, 99.7) for Group A, 75% (95% CI 34.9, 96.8) for Group B, 75% (95% CI 34.9, 96.8) for Group C, 42.9% (95% CI 9.9, 81.6) for Group D, and 0.0% (95% CI 0.0, 45.9) for Group E.

**Figure 3:**
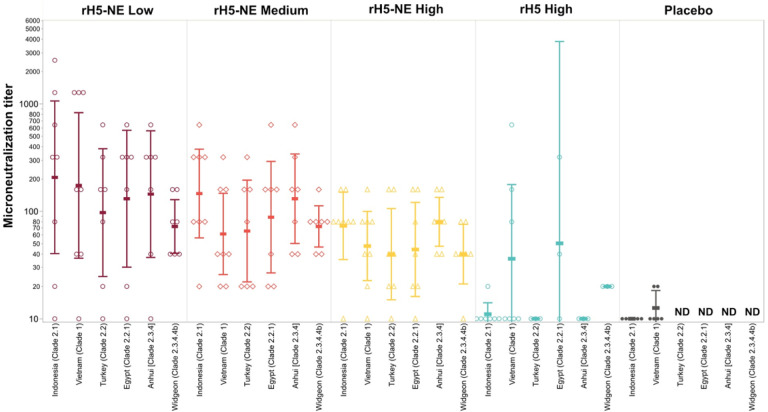
Microneutralization on Day 225 by A/H5N1 virus panel and group Sera from Day 225 (28 days post intramuscular IIV boost) were tested by MN assay using a panel of the following viruses: A/Indonesia/5/2005 (clade 2.1), A/Vietnam/1194/2004 (clade 1), A/Anhui/1/2000 (clade 2.3.4), A/Egypt/3072/2010 (clade 2.2.1), A/Turkey/15/2006 (clade 2.2), and A/Wigeon/sc/22/2021 (clade 2.3.4.4b). Sera that were negative at the initial dilution were assigned a titer of 10. Individual values shown with geometric mean titers indicated by horizontal bar with 95% confidence intervals. Samples from the placebo group were not tested against the broader panel due to low MN titers against the vaccine strains (ND, not done).

We assessed the breadth of protection measured by serum MN at Day 225 against a panel of additional H5N1 viruses, clade 2.2, clade 2.2.1, clade 2.3.4 and clade 2.3.4.4b. (Fig. 3 and **Online display 3**). As we did not assess baseline levels against these H5N1 strains to define seroconversion, we assumed undetectable baseline MN titers. We only performed these additional assays if participants had a measurable MN titer against clade 2.1 and clade 1. rH5-NE containing vaccines elicited seroconversion against clade 2.2 (57.1–71.4%), clade 2.2.1 (57.1–71.4%), clade 2.3.4 (85.7–100.0%), and clade 2.3.4.4b (85.7–100.0%) viruses, in the reverse dose response relationship observed with MN assays against clade 2.1 and clade 1 strains. Group D seroconversion against the panel of viruses was generally lower than the rH5-NE groups.

### Serum immunoglobulin responses.

**Figure 4:**
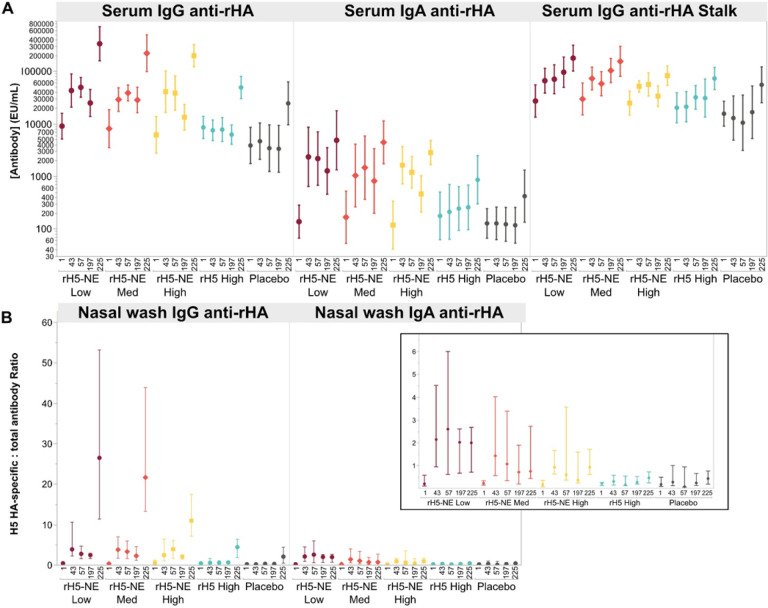
H5N1 clade 2.1 serum and nasal wash binding antibodies by group Panel **A** shows the geometric mean concentration with 95% confidence intervals of H5 A/Indonesia (Clade 2.1)-specific serum IgG and IgA responses, as well as H5 stalk-specific IgG responses (EU/mL). Panel **B** shows nasal wash responses (IgG and IgA) as the median and interquartile range of the ratio of H5-specific IgG or IgA (EU/μg) to total IgG or IgA at each timepoint per group. Inset shows nasal wash responses on an extended y axis.

We measured rH5 (clade 2.1)-specific serum IgG and IgA and H5 stalk-specific serum IgG by ELISA (Fig. 4 and **Online display 4**). At baseline, IgA and IgG levels to rH5 were low, while anti-stalk IgG levels were elevated. Unlike with MN or HAI assays, significant GMT increases (p < 0.05) from baseline were observed for most immunoglobulins measured at Days 57 and 197 in the rH5-NE groups. Significant GMT increases were measured from Day 197 to Day 225 in all groups (p < 0.05) for IgG and IgA, though significant boost responses at this timepoint for H5 stalk-specific serum IgG were only measured in Group C and Group E (p < 0.05).

### Surface plasmon resonance (SPR) binding.

We assessed the quality of humoral immune response using SPR-based real time kinetics assay against both H5N1 clade 2.1 and clade 1 (Fig. 2 **panel C** and **Online display 5**). Antibody binding was significantly higher in the rH5-NE groups than in controls at Day 57 (p = 0.0058). After intramuscular H5N1 IIV, antibody binding increased in each group, with values significantly higher in the rH5-NE groups than in controls at Day 225 (p = 0.0003). Similar patterns were seen with SPR against H5N1 clade 2.1 and against H5N1 clade 1 rHAs, though responses were consistently higher against the rH5 vaccine clade 2.1.

### Antibody-dependent cell-mediated cytotoxicity (ADCC) responses.

**Figure 5:**
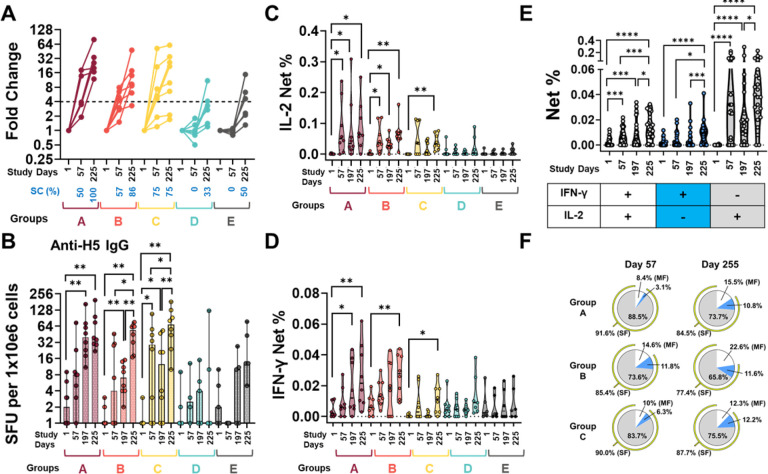
Antibody-dependent cell-mediated cytotoxicity (ADCC), memory B cell and memory T cell responses by group Panel **A** shows the induction of antibodies with ADCC capacity as fold-changes over Day 1. At the bottom of the panel, in blue text, we show the percentage of volunteers that seroconverted (SC) at each timepoint in every group. SC was defined as a 4-fold increase in ADCC titers compared to Day 1 and the dotted line indicates this threshold. Panel **B** shows the frequency of memory B cells producing anti-H5 IgG antibodies, reported as SFU per 1×10e6 cells. The median and 95% CI are shown. Each dot represents an individual. Panel **C** shows the frequency of memory CD4 T cells producing IL-2 (net %) upon ex-vivo stimulation with an H5 peptide pool (A/Vietnam/1203/2004 (clade 1)). **D** shows data from IFN-γ producing cells. In **C** and **D,** the data are shown in violin plots denoting the distribution of the data. Each dot represents one individual. In plots **A-D** data from volunteers vaccinated with the low-dose (Group A), middle-dose (Group B) and high-dose (Group C) of rH5-NE is shown in maroon, orange, and yellow colors. Controls, including the unadjuvanted rH5 (Group D) and placebo (Group E) are shown in cyan and gray colors. Panel **E** shows the ability of rH5-NE (pooled Groups A-Cs) to induce multifunctional (MF) cells at each timepoint of the study. Data are shown in violin plots, and each dot represents one volunteer. MF cells (IL-2+ & IFN-γ+) are shown by the white circles, IFN-γ-only producing cells are shown by the blue circles and IL-2-only producing cells are shown by the grey circles. IL-2-only and IFN-γ-only cells are referred to as Single Functional (SF) cells. Panel **F**displays the frequency of MF and SF cells by Groups A-C at days 57 (post-intranasal vaccination) and 255 (post-systemic boost). The data in panel **D** is presented as percentage of the mean of MF and SF cells. White, blue, and gray areas of the pie show IFN-γ-only, IL-2-only and MF cells, respectively. The yellow semicircle shows the added percentage of SF cells (IL-2-only plus IFN-γ-only). Statistics from panels **B-E** are derived from Wilcoxon signed-rank tests *p<0.05, **p<0.01, ***p<0.005, **** p<0.0001.

ADCC was assessed against rH5 clade 2.1. We defined ADCC seroconversion as ≥ 4-fold increase from baseline. We measured seroconversion in ADCC responses in all rH5-NE groups at Days 57 and 225 (p < 0.05), while comparator groups had antibodies with ADCC activity only at Day 225 (Fig. 5 **panel A** and **Online display 6**). The percentage of participants with ADCC seroconversion at Day 57 was 50% (95% CI 11.8, 88.2) for Group A, 57% (95% CI 18.4, 90.1) for Group B, and 75% (95% CI 34.9, 96.8) for Group C. Seroconversion at Day 225 was 100% (95% CI 54.1, 100) for Group A, 86% (95% CI 42.1, 99.6) for Group B, 75% (95% CI 34.9, 96.8) for Group C, 33% (95% CI 4.3, 77.7) of Group D, and 50% (95% CI 11.8, 88.2) for Group E.

### Mucosal immune responses.

Nasal wash rH5-specific IgG normalized by total IgG was low at baseline for all groups (Fig. 4 **panel B** and **Online display 7**). Significant increases within groups were noted at Days 43, 57, and 197 compared to baseline for all rH5-NE groups (p < 0.05). At Day 225, significantly increased nasal IgG responses were measured for all groups from Day 197 (p < 0.05), with the highest responses in the rH5-NE groups.

**Figure 6:**
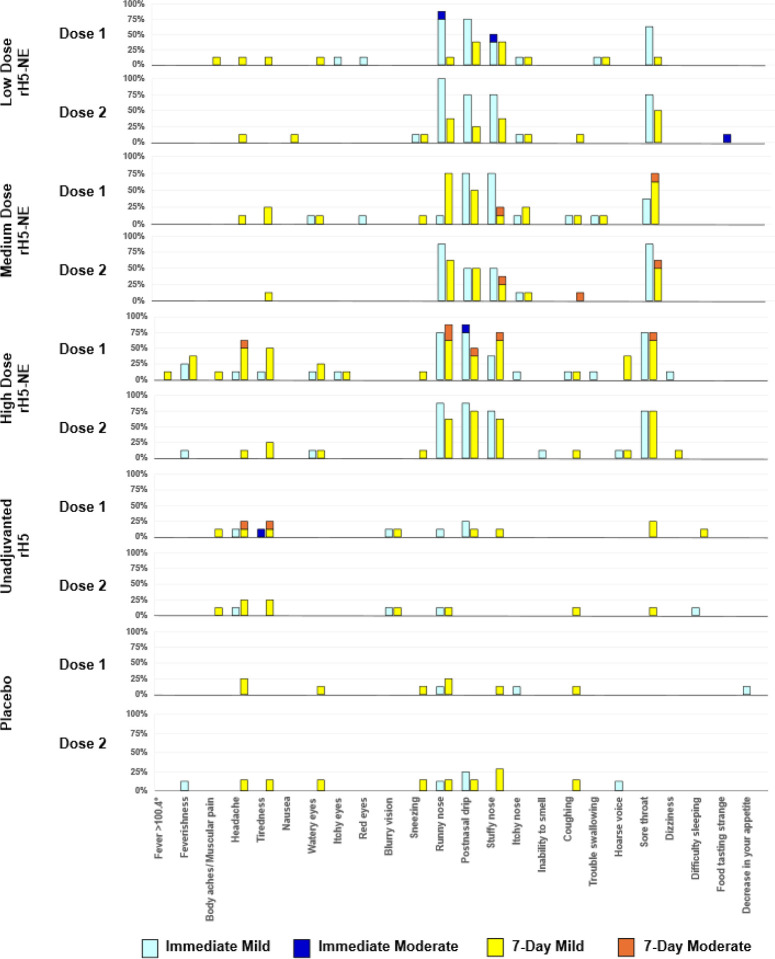
Immediate and seven-day solicited symptoms (excluding immediate events) after the first and second intranasal vaccination by group There were no reports of the following solicited adverse events after intranasal vaccination: bleeding from the nose, difficulty with hearing, double vision, joint pain, ringing in ears, slurred speech, swelling around the eyes, tightness in the chest, or wheezing. There were no events of severity greater than moderate.

Baseline levels of nasal rH5-specific IgA, normalized by total IgA, were low across all groups. However, significant increases were seen in Groups A and B on Days 43, 57, 197, and 225 compared to baseline (p < 0.05), but not for Groups C, D, or E (Fig. 6 **panel B** and **Online display 7**). A significant boost response in nasal IgA levels was seen on Day 225 from Day 197 in Groups A and B (p < 0.05).

### Memory B cells.

We assessed memory B cells with the potential to produce antibodies against rH5 (clade 2.1) in polyclonally expanded PBMC. Analysis of rH5-NE groups showed significant increases in vaccine-specific antibody-secreting cells (ASC) on Day 197 compared to baseline. Only Group C (high-dose rH5-NE) had a significant increase on Day 57 compared to baseline. rH5-NE groups B and C had a significant increase in vaccine-specific ASCs after intramuscular H5N1 IIV on Days 57 and 197 compared to baseline (Day 1) (Fig. 5 **panel B**). The unadjuvanted rH5 group and the comparator groups did not exhibit significant increases in H5-specific ASC from baseline (Day 1) at any subsequent timepoint.

### T cell immunity.

Activated (CD69+) memory CD4 T cells (CD4+, excluding CD45RA + CD62L + cells) were assessed for their ability to produce cytokines or upregulate additional activation markers (e.g., CD154, CD137) after ex-vivo stimulation with rH5 (clade 2.1) (**Online display 8 panel A**) and a H5 peptide pool (clade 1) (Fig. 5).

Peptide stimulations increased IL-2 expression significantly at Days 57, 197 and 225 (compared to baseline) among Groups A and B. Group C increased expression of this cytokine only after the intramuscular H5N1 IIV (Day 225) (Fig. 5 **panel C**). IFN-γ was upregulated by all rH5-NE groups at Day 225. Only Group A had increased expression of this cytokine prior to intramuscular H5N1 IIV (Day 197) (Fig. 5 **panel D**). Memory CD4 T cells stimulated with the rH5 (clade 2.1) had IL-2 and IFN-γ responses of lower magnitude than those identified in cells stimulated with the H5 peptide pool (clade 1), but the trends were similar **(Online display 8 panels C-D).** The comparator groups did not have significant increases from baseline (Day 1) to Day 225 in memory CD4 T cells producing cytokines (Fig. 5 **panels C and D**), as well as in activation or degranulation markers.

Since memory CD4 T cells from rH5-NE groups stimulated with the clade 1 peptide pool had significant expression of IL-2 and IFN-γ, we assessed whether these cells had multifunctional activity, defined as production of more than one cytokine by the same cell. First, we performed these assessments in peptide stimulated cells pooled from all rH5-NE groups (Fig. 5 **panel E**). We identified a significant increase in the frequency of multifunctional cells (IL-2+, IFN-γ+) on Days 57 and 197 compared to baseline. After intramuscular H5N1 IIV vaccination, the frequency of these cells increased significantly. Notably, IL-2 + single functional cells had the highest frequency at every timepoint after intranasal and intramuscular vaccination (Fig. 5 **panel E**). Intramuscular H5N1 IIV increased the frequency of multifunctional memory CD4 T cells in all the rH5-NE groups (shown as percentage of mean multifunctional and single function cells), but the change was highest in Group A and Group B (Fig. 5 **panel F**). For example, in Group A, the frequency of multifunctional cells changed from 8.4% (95% CI 0, 27.1) at Day 57 to 15.5% (95% CI 5.8, 90) by Day 225. We identified no significant production of cytokines or upregulation of CD107a in memory CD8 T cells upon stimulation with H5N1 clade 2.1 or clade 1.

### Safety.

Among participants receiving the first vaccination, immediate (within 60 minutes) reactogenicity symptoms were common and mostly mild (Grade 1) (Fig. 6 and **Supplemental Table 1**). Solicited reactogenicity symptoms occurring in > 5% of participants included runny nose (55.0%), postnasal drip (52.5%), stuffy nose (32.5%), sore throat (35.0%), and itchy nose (10.0%). Participants receiving rH5-NE containing vaccines experienced more local symptoms and three of four moderate severity events (Grade 2). Immediate reactogenicity symptoms after the second dose in > 5% of participants included runny nose (61.5%), postnasal drip (48.7%), sore throat (48.7%), stuffy nose (41.0%), itchy nose (5.1%), feverishness (5.1%), headache (5.1%), watery eyes (5.1%). Most symptoms occurred in rH5-NE groups, including one moderate severity event (Grade 2). Solicited 7-day post-vaccination reactogenicity symptoms were mostly mild (Grade 1), remained more common in rH5-NE groups, and were similar to the immediate reactogenicity profiles.

Five related unsolicited adverse events within 1 hour of vaccination were mild, including elevated diastolic blood pressure, toothache, sinus pain, and nasal discomfort (2 participants) (**Supplemental Table 2 and Supplemental Table 3**). Two solicited events (mild cough and postnasal drip) began during the 7-day post-vaccination period and extended beyond it, qualifying as unsolicited events. All adverse events were self-limited. There were two other related unsolicited adverse events (cough and upper-airway cough) within the 28-day post-vaccination period, and none of the related unsolicited adverse events was medically attended. No adverse events after either intranasal vaccination were of severe or higher severity.

Laboratory abnormalities within seven days post-dose 1 and 14 days post-dose 2 were mostly mild (Grade 1) (**Supplemental Table 2 and Supplemental Table 4)**. Two moderate abnormalities (Grade 2) occurred after the first vaccination with no severe or higher severity events. There were no moderate or greater severity adverse events after the second vaccination.

The licensed H5N1 IIV was well tolerated with few solicited adverse events and no related unsolicited events. Two participants had unrelated Grade 3 low hemoglobin, assessed as due to study phlebotomy. No other moderate or greater severity events occurred after H5N1 IIV (**Supplemental Table 4** and **Supplemental Table 5**)

Throughout the trial there were no potentially immune-mediated medical conditions, new onset chronic medical conditions, or serious adverse events (**Supplemental Table 6**).

## DISCUSSION

In this study, we evaluated the safety and immunogenicity of a novel intranasal adjuvanted influenza A/H5 vaccine. As previous avian influenza LAIV studies had demonstrated vaccine-induced immune responses were evident only after a subsequent intramuscular inactivated vaccine boost^12,14,15^, we incorporated a heterologous H5N1 IIV (A/Vietnam/1203/2004, clade 1) vaccine dose into the trial six months after the two rH5-NE doses (A/Indonesia/05/2005, clade 2.1). Like those previous studies, we observed low HAI and MN responses after the primary vaccination series but rapid, robust responses after intramuscular boost^12–15^. Additionally, the rH5-NE groups elicited high neutralizing antibody titers against diverse H5N1 virus clades.

The immune responses observed after the intramuscular H5N1 IIV boost indicate a strong recall response in groups that received the intranasal rH5-NE vaccines. Seroconversion rates following H5N1 IIV in the rH5-NE groups ranged from 38–100% for the H5N1 clade 1 virus, surpassing the 19–26% seroconversion rates after a single-dose of H5N1 IIV in a previous study^20^. The rapid HAI responses detected seven days after the H5N1 IIV boost, along with differential immune responses at 28 days post-boost in the intranasal rH5-NE groups, support a recall response of cross-reactive memory B cells rather than a primary response to the intramuscular vaccination.

We observed a reverse dose response to the rH5-NE vaccine in several immune assays, a phenomenon previously noted in adjuvanted intramuscular vaccines for avian influenza^21,22^. This finding may suggest that increased antigen doses may activate extrafollicular B cells with lower affinity, which are not targeted to the germinal centers for further affinity maturation. Such B cells are more likely to produce non-neutralizing antibodies upon boosting^23,24^. An alternative possibility is that larger antigen doses with the NE adjuvant favor the activation of CD4 regulatory T cells (Tregs) over Effector Memory CD4 T cells, with activated Tregs suppressing immune responses^25^. Some of the CD4 T cell responses we assessed showed a similar reverse dose response pattern, though it is unclear whether the frequencies of T follicular helper cells (essential for germinal center seeding and somatic hypermutation) would also follow this pattern. Of note, ADCC-mediating antibodies followed an increasing dose response, suggesting a disassociated evolution of antibodies with different functions.

The rH5-NE vaccines were well-tolerated. Mild local nasal symptoms were common, occurred more frequently in the adjuvanted vaccines than the comparators, and were often reported within 60 minutes of vaccination. While rH5-NE reactogenicity was minimal in preclinical studies and our trial, we remain cautious given the history of Bell’s palsy associated with an intranasal split-virus influenza vaccine adjuvanted with *E. coli* heat-labile toxin^26^. While the cause was likely related to the specific adjuvant^27^, which was more immunostimulatory than the nanoemulsion used in our trial, future studies of adjuvanted intranasal vaccines should monitor for this adverse event closely.

Our study suggests that the absence of HAI or MN responses following the primary intranasal series may reflect immunological compartmentalization, with priming potentially focused on the upper respiratory tract. We saw increases in both nasal IgG and IgA levels after the intranasal vaccination series, but low magnitude nasal IgA boost after H5N1 IIV, further supporting this notion. It remains unclear whether the nasal wash immunoglobulins originated from local production or were due, at least in part, to extravasation from the vasculature. Attempts to investigate this by isolating mononuclear cells from nasal wash samples for flow cytometry were unsuccessful due to insufficient cell yields.

Despite these limitations, immunogenicity signals elicited by the primary intranasal rH5-NE series, including significant serum and mucosal IgG and IgA responses, SPR binding antibodies, and ADCC activity, warrant further investigation. ADCC, in particular, has been historically underappreciated; however recent studies have demonstrated that antibodies with ADCC capacity that target conserved regions of HA are cross-reactive and can be protective in adoptive transfer models^28^. Additionally, within the framework of the European Union’s Innovative Medicines Initiative-funded project “FLUCOP,” ADCC assays have been shown to be standardized, cost-effective, and viable as an alternative correlate of influenza protection^29^. In our study, robust ADCC activity was detected across all rH5-NE groups following intranasal vaccination, suggesting that ADCC assays could serve as a valuable tool for evaluating mucosal vaccine priming. However, this hypothesis requires confirmation in larger, future studies.

The separate rH5-NE vaccine components have been evaluated in human trials previously. A 2010 randomized trial tested the nanoemulsion adjuvant combined with seasonal inactivated influenza vaccine at doses of 4–10 μg in a single intranasal dose^30^. Compared to the approved intramuscular vaccine and an intranasal placebo, 28-day HAI seroconversion ranged from 0–25% for intranasal groups and 60–80% for the intramuscular group. Vaccine-specific IgA levels were similar across all groups. A 2011 trial of intramuscular rH5 in a dose-ranging study (15–90 μg, with/without Alhydrogel adjuvant) found 10% seroconversion after the second dose in the best-performing group^16^.

Given prior clinical experience with the nanoemulsion adjuvant and rH5 antigen, the intranasal rH5-NE formulation’s performance in our study is noteworthy. In the 2010 trial, serum HAI titers were not expected to correlate with intranasal vaccine performance, and prior exposure to seasonal influenza viruses likely confounded immune response assessments. For the intramuscular rH5 study, inactivated avian influenza vaccines are known to have low immunogenicity, often requiring high antigen doses or potent adjuvants to induce measurable HAI responses^31^. These studies highlight challenges in influenza vaccine development, including unclear immunogenicity measures of success for mucosal vaccines, confounding by pre-existing immunity, and challenges advancing novel H5N1 prevention technologies.

The influenza A/H5N1 strains in the rH5 antigen (A/Indonesia/05/2005, clade 2.1) and H5N1 IIV (A/Vietnam/1203/2004, clade 1) were isolated nearly 20 years before our trial. Since then, H5N1 viruses have evolved substantially, including the emergence of clade 2.3.4.4b, which has caused widespread infections in poultry, livestock, and over 50 sporadic human cases in North America in 2024 and a recent death^32,33^. To assess cross-protective potential, we performed MN assays against a panel of H5N1 strains, including the vaccine clades and clade 2.3.4.4b. All rH5-NE groups attaining the highest neutralization titers elicited cross-protective MN responses. Similar cross-protection against H5N1 clade 2.3.4.4b has been shown with licensed adjuvanted H5N1vaccines^34^. These findings suggest the rH5-NE vaccine has potential as a preventive intervention. The nanoemulsion adjuvant’s ability to elicit strong memory immune responses at low antigen doses may reduce the required vaccine dose and help expand the available supply of avian influenza vaccines.

Our trial has notable strengths, including extensive immunological assessments of cell-mediated, humoral, and mucosal immune responses, with consistent patterns across varied readouts and signs of mucosal immune priming that warrant further investigation. The intramuscular H5N1 IIV vaccine used to probe rH5-NE priming revealed immunological activity that otherwise would not have been detected. This heterologous prime-boost approach, previously used in H5N1 trials, elicits broader neutralizing and anti-stalk antibody responses compared to homologous regimens^35–39^. The rH5-NE vaccine provides an excellent model for advancing mucosal immune priming research. It was well-tolerated, and the absence of pre-existing H5N1 immunity in the general population, simplified immune response interpretation. Additionally, its recombinant design avoids the safety concerns associated with H5N1 LAIV trials which required quarantine given risks of recombination with seasonal influenza viruses^14,40^.

Our study has several limitations. As a first-in-human trial, there were no pre-specified hypothesis tests, and the sample size was not chosen to detect group differences with sufficient statistical power. Improvements in mucosal sampling and analysis are also necessary. Variability in nasal wash volumes complicates data interpretation. Standardization of mucosal sampling could improve immune evaluation consistency and comparisons across studies. Absent advances in direct measurements of mucosal immunity, this study demonstrates the value of including an intramuscular boost to uncover mucosal priming for novel pathogens. Trial designs for evaluating mucosal vaccines against endemic respiratory diseases will need to account for preexisting immunity.

Our phase I trial demonstrates that an adjuvanted recombinant H5 influenza vaccine administered intranasally elicits immune priming against a diverse panel of H5N1 viruses. The results highlight the need for correlates of protection specific to mucosal influenza vaccines. Promising immune priming signals position this vaccine as a valuable tool for studying biomarkers of mucosal vaccine performance. As global efforts to develop mucosal vaccines accelerate to improve protection against respiratory virus infection and transmission, our trial underscores both the opportunities and challenges ahead.

## ONLINE METHODS

We conducted this study at the University of Maryland School of Medicine Center for Vaccine Development and Global Health (Baltimore, MD, USA). Healthy, non-pregnant, non-lactating adults aged 18 to 45 years were included. Participants were recruited from a volunteer database at the clinical site and through advertisements in the Baltimore/Washington, DC, region. Those meeting all eligibility criteria—including screening lab tests, negative pregnancy tests (when applicable), medical history, and physical exam—within 28 days before the first vaccination were eligible for enrollment. Individuals with chronic medical conditions or medications affecting safety or study endpoints were excluded.

Healthy adult participants were randomized into five groups: three groups received rH5-NE at escalating antigen levels (25 μg, 50 μg, and 100 μg), one group received unadjuvanted rH5 (100 μg), and one group received a formulation buffer placebo. Participants were enrolled and randomized sequentially in a step-wise, dose-escalation process using four vaccination cohorts. Each rH5-NE dose level was assessed in two sentinel participants before subsequent doses were administered.

Intranasal vaccinations were given on Days 1 and 29. On Day 197, all participants received a heterologous intramuscular boost with a 90-μg dose of licensed, inactivated influenza H5N1 vaccine (H5N1 IIV). Local and systemic immune responses were measured, and vaccine safety was assessed. Participants were followed through Day 393 for safety and immunological endpoints.

The primary objective of the study was to evaluate the safety and reactogenicity of rH5-NE. Secondary objectives included assessing mucosal immune responses (IgA, IgG, and T-cell mediated immunity [T-CMI]) and humoral immune responses (serum HAI) after two doses of rH5-NE, and the safety and reactogenicity of a single booster dose of intramuscular H5N1 IIV.

Pre-specified exploratory objectives were as follows:

1. To assess humoral immune responses to rH5-NE and H5N1 IIV vaccine-specific antigens at multiple timepoints.
2. To evaluate primary T-CMI in peripheral blood to rH5-NE and H5N1 IIV vaccine-specific antigens.
3. To measure memory B-cell responses by ELISpot in peripheral blood to rH5-NE and H5N1 IIV vaccine-specific antigens.

### Study vaccines.

The rH5-NE vaccine consisted of three components: rH5 glycoprotein antigen, 60% W_80_5EC nanoemulsion adjuvant, and a proprietary formulation buffer^17,18,41^. The antigen was a recombinant hemagglutinin (HA) glycoprotein expressed in the tobacco plant *Nicotiana benthamiana*, produced by Fraunhofer USA Center for Molecular Biotechnology (Plymouth, MI, USA), lot CMB-BDS-0100–017. It was a fusion protein containing:

1. HA from derived from A/Indonesia/05/2005 (H5N1, clade 2.1),
2. A poly-histidine tag (6xHis) to enhance purification efficiency, and
3. The endoplasmic reticulum retention signal (KDEL) for efficient protein expression.

The 60% nanoemulsion adjuvant (W_80_5EC) was manufactured by BlueWillow Biologics, Inc. (Ann Arbor, MI) under GMP conditions using high shear homogenization of water, ethanol, cetylpyridinium chloride, Tween-80, and highly refined soybean oil to form an oil-in water nanoemulsion with a mean particle size of ~400 nm.

The final vaccine consisted of 20% W_80_5EC, achieved by mixing 60% W_80_5EC with rH5 and formulation buffer on-site. Two comparator groups received intranasal vaccines: one with unadjuvanted rH5 mixed with formulation buffer and the other with formulation buffer alone. After completing the intranasal vaccine primary series, all groups received the Influenza Virus Vaccine, H5N1 (H5N1 IIV), produced by Sanofi (Swiftwater, PA, USA), lot #U2147A/UD08916. This licensed, split-virion vaccine, derived from A/Vietnam/1203/2004 (H5N1, clade 1), is approved for intramuscular administration as a two-dose series for persons aged 18 to 64 years at increased risk of exposure to the H5N1 influenza virus subtype contained in the vaccine^19^. Manufactured in 2004–2005, it contains 90 μg HA per 1 mL dose. Stability testing conducted in August 2022 confirmed it met manufacturer stability specifications.

### Procedures.

Participants provided written informed consent before any data collection or study procedures. Eligible participants were enrolled, randomized to a group, and vaccinated. The randomization list for the two intranasal vaccinations was prepared by an unmasked study statistician using block randomization for each cohort. If all participants proceeded to the second vaccination, the final vaccine allocation ratio would be 1:1:1:1:1.

For the intranasal vaccinations, participants and study staff involved in post-vaccination assessments were blinded to vaccine allocation. However, due to differences in the appearance of rH5-NE compared to unadjuvanted or placebo formulations, the study staff administering the vaccines were unmasked. These staff members managed vaccine accountability, storage, preparation, and administration but were not involved in subsequent study assessments. The third vaccination, using H5N1 IIV for all participants, was conducted in an open-label manner.

Intranasal vaccination was performed with a metered electronic pipette, administering ten successive 25 μL droplets onto bilateral inferior turbinates, for a total dose of 500 μL. Intramuscular H5N1 IIV was administered in the preferred deltoid, following manufacturer guidelines^19^. After vaccination, participants were observed in the study clinic for 60 minutes (after intranasal vaccines) or 30 minutes (after H5N1 IIV). Participants had vital signs recorded, underwent examination by a study clinician (a required procedure after intranasal vaccinations and performed only if needed for H5N1 IIV), completed an immediate reactogenicity assessment, received electronic symptom diary training, and were discharged.

Participants were contacted by phone three days after each vaccination to assess adverse events (AEs), reinforce electronic diary instructions, and remind them of their next appointment. Participants returned to the clinic after seven days (for the first and third vaccinations) and 14 days (for the second vaccination) for safety assessments on Days 8, 43, and 204. At these visits, a study investigator reviewed electronic diaries, evaluated vaccine reactogenicity, and assessed other safety events. Safety hematology and chemistry lab evaluations were performed on venous blood samples at these visits.

An independent Safety Monitoring Committee (SMC) convened after seven days of safety data were collected for each of the first three cohorts. The SMC applied standardized rules to determine whether the study could proceed with vaccination of the remainder of participants at the same intranasal vaccine dose level and additional participants at the next intranasal vaccine dose level.

In-person safety and immunogenicity assessments were conducted on Days 57 and 225. Each visit included standardized neurologic, otorhinoscopic, and respiratory examinations. Blood samples (70 mL) for peripheral blood mononuclear cell (PBMC) isolation were collected on Days 1, 43, 57, 197, and 225. Serum samples (17 mL) were collected on Days 1, 29, 43, 57, 197, 204, and 225, and nasal wash samples (3–7 mL) were obtained at screening and on Days 8, 43, 57, 197, 204, and 225. These specimens were evaluated for vaccine immune responses. Safety phone calls were conducted on Day 90 and at trial close-out (Day 393). Data collection was managed using an electronic Case Report Form.

### Safety assessments.

We assessed vaccine reactogenicity based on the occurrence of solicited local and systemic reactions from the time of each vaccination through seven days post-vaccination. Reactogenicity assessments varied by the route of administration:

- Intranasal rH5 Vaccinations: Solicited local reactions included watery eyes, itchy eyes, red eyes, blurry vision, double vision, swelling around the eyes, sneezing, runny nose, postnasal drip, stuffy nose, itchy nose, inability to smell, bleeding from the nose, coughing, difficulty with hearing, ringing in ears, tightness in the chest, wheezing, trouble swallowing, hoarse voice, sore throat, slurred speech, dizziness, difficulty sleeping, food tasting strange, and decrease in appetite. Solicited systemic reactions included fever (>38° C), feverishness, joint pain, body aches/muscular pain, headache, tiredness, and nausea.
- Intramuscular Vaccinations: Solicited local reactions included pain, tenderness, erythema/redness, and induration/swelling. Solicited systemic reactions included nausea/vomiting, diarrhea, headache, fatigue, and myalgia.

Unsolicited non-serious AEs and medically attended AEs were recorded from the time of each vaccination through 28 days post-vaccination. Serious adverse events (SAEs), new-onset chronic medical conditions (NOCMCs), and potentially immune-mediated medical conditions (PIMMCs) were monitored for 12 months following the final intranasal vaccination (through Study Day 393). Safety laboratory AEs and unsolicited AEs were assessed for severity, clinical significance, and causality (related or unrelated) by a study clinician using standardized grading criteria.

### Immunogenicity assessments.

We assessed immune responses by several assays at multiple timepoints throughout the study.

Mucosal immune evaluations included:

- H5-specific clade 1-specific and total IgA and IgG by ELISA.

Humoral immune evaluations included:

- HAI for H5N1 A/Vietnam/1203/2004 (clade 1) and A/Indonesia/05/2005 (clade 2.1) at baseline (Day 1) and Days 57, 197, and 225. A post hoc analysis included HAI for H5N1 A/Indonesia/05/2005 (clade 2.1) at Day 204.
- ELISAs for H5 A/Indonesia/05/2005 (clade 2.1) -specific IgG and IgA and H5 stalk IgG at baseline (Day 1) and Days 43, 57, 197, and 225.
- Post hoc analyses of viral microneutralization (MN) for H5N1 A/Vietnam/1203/2004 (clade 1) and A/Indonesia/05/2005 (clade 2.1) at baseline (Day 1) and Days 57 and 225, as well as MN for H5N1 A/Turkey/15/2006 (clade 2.2), A/Egypt/3072/2010 (clade 2.2.1), A/Anhui/1/2000 (clade 2.3.4), and A/Wigeon/SC/22/2021 (Clade 2.3.4.4b) Day 225 only.
- Post hoc analyses of surface plasmon resonance (SPR) binding for H5N1 A/Vietnam/1203/2004 (clade 1) and A/Indonesia/05/2005 (clade 2.1) rHA proteins at baseline (Day 1) and Days 57 and 225.
- Post hoc analyses analysis of antibody-dependent cell-mediated cytotoxicity (ADCC) for A/Indonesia/05/2005 (clade 2.1) at baseline (Day 1) and Days 57 and 225.

Cellular immune evaluations included:

- T cell immune responses:
  - Activation markers (e.g., CD69, CD137 and CD154), cytokines/chemokines, and degranulation markers in CD4 and CD8 T cell memory subsets by flow cytometry for H5N1 clade 2.1 at baseline (Day 1) and Days 57, 197, and 225. Post hoc analyses included the same assays for H5N1 clade 1.
- Memory B cell immune responses:
  - H5-specific memory B cells measured by ELISpot for H5N1 clade 2.1 at baseline (Day 1) and Days 57, 197, and 225.

### Total and antigen-specific IgG and IgA ELISAs.

For measurements of total IgA or IgG, microplates were coated with 0.5 μg/mL of purified anti-IgA (α-chain specific) or anti-IgG (γ-chain specific; Jackson Immunoresearch -JIR-, PA) overnight at 4°C. For the detection of antigen-specific IgA or IgG, plates were coated with 2 μg/mL of plant-derived A/Indonesia/5/05/2005 (H5N1; clade 2.1) rH5 (Fraunhofer USA Center for Molecular Biotechnology) or with 0.5 μg/mL of headless H5 stalk- based on H5: A/Indonesia/05/05 as described previously^42^ (produced in-house). After coating, plates were washed with PBS containing 0.05% Tween 20 (PBST) and blocked with PBST containing 5% nonfat dry milk (PBSTM) for 1 h at 22°C. Nasal wash samples were diluted 1:1,000 and 1:5,000 in PBSTM for total IgA and total IgG measurements and 1:5 for rH5-specific IgA and IgG.

Serum samples were diluted 1:10,000 for quantification of rH5-specific IgA and IgG, and 1:2,000 for H5 stalk-specific IgG. Plates were incubated with the samples for 2 h at 22°C, washed with PBST, and incubated with biotinylated goat anti-human Fc-specific IgA or IgG (JIR, PA) diluted 1:5,000 in PBSTM for 1 hour at 22°C.

After washing, TMB substrate was added to the plates and allowed to develop for 1–15 minutes at room temperature (in the dark, with shaking). The reaction was stopped by adding 100 μL/well of 1 M phosphoric acid (Sigma). Antibody concentrations determined through interpolation of Absorbance values from standard curves.

### Hemagglutination inhibition (HAI) assay.

The HAI assay was performed following a standardized protocol^43^. Influenza virus was propagated from A/Indonesia/05/05, a H5N1-E4, PR8-backboned vaccine seed virus or A/rgVN/1203/2004, DYE2, a reverse-genetics engineered vaccine seed virus, both provided by the Food and Drug Administration Center for Biologics Evaluation (FDA/CBER). A solution of 0.5% horse red blood cells (RBCs) was used for improved sensitivity to H5N1 viruses. Virus potency was confirmed by back-titration. Serum-only and RBC-only controls were included along with positive controls (a human polyclonal reference antisera to Influenza A/Indonesia/05/2005, BEI Resources NR-33668 and the WHO International Standard for antibody to influenza H5N1 virus, NIBSC 07/150 for A/Vietnam/1203/2004). All serum samples were treated with receptor-destroying enzyme (RDE) prior to testing and then serially diluted in 96-well U-bottom plates. Influenza virus stocks, stored at −80°C, were thawed immediately before use and the viral hemagglutination unit adjusted to 8 units in PBS, pH 7.4. The virus was then added to the sample-containing wells at a 1:1 ratio, and plates were incubated for 30 min at room temperature. Subsequently, 0.5% RBCs were added to each well and the plates incubated for 60 min at room temperature. Antibody titers were determined by the CypherOne HAI plate reader (InDevR, software version 4.0.0.19)^44^ as well as visually assessing the plates for hemagglutination. The antibody titer was defined as the reciprocal of the highest serum dilution that contains an RBC agglutination inhibition precipitation pattern that is similar in size, well clarity, and morphology to the positive serum control.

### Antibody titer determination by microneutralization (MN) assay.

Vaccine-induced antibody titers were assessed by virus MN assays^45^. MN titers assessed viral-neutralizing activity in MDCK cells based on the methods of the pandemic influenza reference laboratories of the Centers for Disease Control and Prevention (CDC), with minor modifications provided in an updated protocol issued by the CDC. MN titers were measured against H5N1 vaccine strains of A/Vietnam/1194/2004 (clade 1), A/Indonesia/5/2005 (clade 2.1), A/Anhui/1/2000 (clade 2.3.4), A/Egypt/3072/2010 (clade 2.2.1), A/Turkey/15/2006 (clade 2.2), and A/Wigeon/sc/22/2021 (clade 2.3.4.4b). These vaccine strains consist of the relevant HA and NA on the PR8 backbone and with the HA polybasic cleavage site removed. All MN assays were conducted at BSL-2.

Sera were tested at an initial dilution of 1:20, and those that were negative (<1:20) were assigned a titer of 10. The titers represent the dilution of serum with highest dilution that completely suppressed virus replication. All sera were tested in triplicate, and the geometric mean value was used for analysis. The MN assays were performed at least twice independently for each serum sample.

### Binding antibody measurements by Surface Plasmon Resonance (SPR).

Steady-state equilibrium binding of post-H5N1 vaccinated human sera was monitored at 25 °C using a ProteOn SPR biosensor (Bio-Rad)^45–47^. The recombinant HA globular domain (rHA1-His_6_) for the A/Indonesia/05/2005 (clade 2.1) or from H5N1- A/Vietnam/1203/2004 (clade 1) influenza virus strain was coupled to a GLC sensor chip with amine coupling with 1,000 RU in the test flow cells. Samples of 200 μl of sera at 10-fold dilutions were injected at a flow rate of 50 μl min^−1^ (120-s contact time) for association, and disassociation was performed over a 600-s interval. Responses from the protein surface were corrected for the response from a mock (no coating) surface and for responses from a separate, buffer-only injection. Binding antibodies were determined from two independent SPR runs.

### Memory B cell assay.

Cryopreserved PBMC were thawed^48,49^ and polyclonally expanded (5–6 days) as described before^49–52^. Expanded PBMC were harvested, counted, and seeded in quadruplicate wells (250,000 cells per well) of multi-screen plates coated with rH5 from A/Indonesia/5/2005 (clade 2.1) (Fraunhofer USA Center for Molecular Biotechnology) at 3 μg/mL. Controls included wells coated with 1) goat anti-human IgA (Total IgA control; 5 μg/mL) (JIR, PA); 2) goat anti-human IgG (Total IgG control; 5 μg/mL) (JIR, PA); or 3) 1x PBS. In Total IgG and IgA control wells, 2-fold dilutions of the cells, starting at 24,000, were seeded in duplicate^50–53^. 5 hours later the plates were washed and incubated with goat-anti-human IgA-HRP (JIR, PA) or goat-anti-human IgG-HRP (JIR, PA). IgG and IgA Spot Forming Cells (SFC) were visualized with AEC substrate. Total and antigen-specific B memory SFC were calculated as SFC/10e6 cells.

### Assessment of cytokine production by T cells.

Cryopreserved PBMC were thawed and rested for 4 hours (37°C, 5% CO_2_). PBMC were then washed and partitioned into four 2×10e6 cell aliquots. Two aliquots were stimulated with 1) rH5 from A/Indonesia/5/2005 (clade 2.1) (Fraunhofer USA Center for Molecular Biotechnology) at 3 μg/mL; and 2) a H5 HA peptide pool from A/Vietnam/1203/2004 (clade 1) at 2 μg/mL of each peptide. The peptide pool consisted of a 93-peptide array (12- or 17-mers, with 11aa overlap) from BEI Bioresources (NR-18974). The other two aliquots served as negative (media) and positive (Staphylococcal enterotoxin B -SEB-; 10 mg/mL) controls. All samples received anti-CD107a-FITC (clone H4A3; Becton Dickinson Biosciences -BDB-) and anti-CD28/CD49d co-stimulatory antibodies (BDB, USA). Two hours later, Brefeldin A and Monensin were added and incubated overnight (16 hours, at 37°C, 5% CO_2_). Next day the cells were stained for flow-cytometry^48,54–57^. Briefly, cells were stained for viability (fixable yellow staining dye; 20 min; RT) and then with a surface antibody cocktail (30 min, RT) that included: CD62L-PE (clone DREG; eBiosciences), CD4-PerCP-Cy5.5 (clone: L200; BDB), CD19-BV570 (clone: HIB19; BioLegend), CD56-BV570 (clone: HCD56; BioLegend), CD3-BV650 (clone: SK7 BDB), CD8-A700 (RPA-T8; BDB), CD45RA-APC-H7 (clone: HI100; BDB). The cells were then fixed, permeabilized and stained with an intracellular cocktail (30 min, RT) including the next antibodies: CD69-ECD (clone: TP1.55.3; BC), IFN-γ-PE-Cy7 (clone: B27; BDB), IL-17A-BV605 (clone: BL168; BioLegend), TNF-α-BV711 (clone: MAb11; BioLegend), CD154-BV785 (clone: 24–31; BioLegend), IL-2-APC (clone: MQ1–17H12; BioLegend), and CD137-BV421 (clone: 4–1BB; BioLegend). Cells were then fixed (1% PFA) and the samples collected in a custom LSRII flow cytometer (BD, USA). FCS files were analyzed using FlowJo (Tree Star, San Francisco, USA).

### Antibody-dependent cell-mediated cytotoxicity (ADCC) assay.

rH5 from A/Indonesia/5/2005 (clade 2.1) (Fraunhofer USA Center for Molecular Biotechnology) was biotinylated (Abcam, USA) and then used to coat polystyrene-streptavidin beads (SA-beads; Spherotech, USA). For the assay, each reaction used 5 μL of SA-beads coated with ~250 ng of rH5. Plasma from vaccinated volunteers (25 μL per reaction) was incubated with rH5-SA-beads (overnight, 4°C), the beads were subsequently washed 2x with assay media (cRPMI with 10% ultra-low IgG FBS), resuspended in 50 μL, and added into a 96-well plate (50 μL per well). 1.5×10e6 Jurkat cells expressing the firefly luciferase gene under the control of NFAT response elements and constitutively expressing CD16a (V158) (BPS Bioscience) were added to the beads (50 μL) and incubated for 5 hours (37°C; 5% CO_2_). The luciferase signal was detected by a luminometer after cell lysis and the addition of luciferin substrate (BPS Bioscience). The assay was performed in triplicate wells.

### Statistical analysis.

There were no pre-specified hypothesis tests for this phase I trial. The sample size of 40 with 8 persons per vaccine group was chosen without the intention that group differences would be detected with a sufficient power but was consistent with FDA guidance^58^.

The analysis population includes all participants who received study vaccination and for whom data were available at any particular timepoint. Outcome data were analyzed by vaccine group. Demographic and medical data were summarized using descriptive statistics. Safety endpoints were tabulated by severity and relatedness. Mucosal IgG and IgA were summarized by ELISA units, fold rise, and whether ≥4-fold rise was achieved. Given the low cell yields, mucosal T cell responses were not analyzed. HAI, MN, and ELISA data were summarized by Geometric Mean Titer (GMT), geometric mean fold rise (GMFR), and proportion of seroconversion. We defined antibody seroconversion as either a pre-vaccination titer <10 and a post-vaccination titer ≥40 or a pre-vaccination titer ≥10 and a minimum of four-fold rise in post-vaccination titer. In post hoc analyses, when there was no baseline titer information, we defined seroconversion as a post-vaccination titer ≥40, and we defined seroconversion as ≥4-fold rise over baseline ADCC analysis.

For binary outcomes such as presence/absence of an AE or seroconversion (Yes/No), proportion was calculated for each group with the corresponding 95% confidence intervals. At each time point of interest, the difference in proportions between the vaccine groups was compared using Fisher’s exact test or Chi-square test as appropriate; and the difference in continuous outcomes of interest between the groups were compared using Kruskal-Wallis H-test. Pairwise comparisons were conducted when needed. Wilcoxon signed-rank test was used to compare differences in antibody titers between time points within each vaccine group. No adjustments were made for multiple comparisons. All statistical analyses were performed using Stata/SE version 18 (Stata Corp, College Station, USA), and all figures were generated using Microsoft Office software, GraphPad Prism (v.10) (Boston, MA), and JMP (v.18, SAS institute, Cary, NC).

### Regulatory and ethics.

The protocol and informed consent forms were reviewed and approved by the University of Maryland, Baltimore Institutional Review Board and the study was registered at Clinicaltrials.gov (NCT05397119).
